# Status of the largest extant population of the critically endangered Aeolian lizard *Podarcis raffonei* (Capo Grosso, Vulcano island)

**DOI:** 10.1371/journal.pone.0253631

**Published:** 2021-06-23

**Authors:** Gentile Francesco Ficetola, Iolanda Silva-Rocha, Miguel A. Carretero, Leonardo Vignoli, Roberto Sacchi, Andrea Melotto, Stefano Scali, Daniele Salvi

**Affiliations:** 1 Department of Environmental Science and Policy, Università degli Studi di Milano, Milano, Italy; 2 CNRS, Laboratoire d’Ecologie Alpine (LECA), Univ. Grenoble Alpes, Grenoble, France; 3 CIBIO, Centro de Investigação em Biodiversidade e Recursos Genéticos, Universidade do Porto, InBio Laboratório Associado, Vairão, Portugal; 4 Departamento de Biologia, Faculdade de Ciências da Universidade do Porto, Porto, Portugal; 5 Dipartimento di Scienze, Università Roma Tre, Rome, Italy; 6 Department of Earth and Environmental Sciences, University of Pavia, Pavia, Italy; 7 Centre of Excellence for Invasion Biology, Department of Botany and Zoology, Stellenbosch University, Stellenbosch, Western Cape, South Africa; 8 Museo di Storia Naturale di Milano, Milano, Italy; 9 Department of Health, Life and Environmental Sciences, University of L’Aquila, Via Vetoio, L’Aquila, Italy; State Museum of Natural History, GERMANY

## Abstract

The Aeolian wall lizard *Podarcis raffonei* is an island endemic that survives only on three tiny islets, and on the Capo Grosso peninsula of the Vulcano island, thus is among the European vertebrates with the smallest range and one of the most threatened by extinction. This species is declining due to competition and hybridization with the non-native lizard *Podarcis siculus*, but a regular monitoring program is lacking. Here we assessed the size and status of the Capo Grosso population of *P*. *raffonei* on Vulcano. In September 2015 we captured 30 individuals showing the typical brown phenotype of *P*. *raffonei*, while one single male showed a green phenotype, apparently intermediate between *P*. *raffonei* and the non-native *Podarcis siculus*. In May 2017, only 47% of 131 individuals showed the typical brown phenotype (*P*. *raffonei*-like) and 53% showed the green phenotype (*P*. *siculus*-like). Based on *N*-mixture models and removal sampling the estimated size of the Capo Grosso population was of 800–1300 individuals in 2017, being similar to 2015; available data suggest that the total range of the species could be as small as 2 ha. The frequency of individuals with the typical *P*. *raffonei* phenotype dramatically dropped between two samplings with a parallel increase of individuals displaying the green phenotype. Observation on outdoor captive-bred individuals demonstrates plasticity for colouration in *P*. *raffonei* individuals from Capo Grosso, with several individuals showing the typical brown pattern in September 2017 and a green pattern in March 2021. Non-exclusive hypotheses, including hybridization with *P*. *siculus* and plasticity in colour pattern of *P*. *raffonei*, are discussed to explain the phenotypic shifts of the *P*. *raffonei* population of Capo Grosso. While genomic evidence is required to reach conclusions and investigate eventual hybridization, it is urgent to undertake a programme for the monitoring and management of this lizard.

## Introduction

Monitoring of populations is an essential conservation task. Frequent monitoring is necessary to determine natural fluctuations, to detect declining trends, to ascertain the conservation status of populations and species and to ensure the effectiveness of eventual management measures [1–4]. This is recognised by the Habitats Directive of the European Union (Directive 92/43/EEC), which requires that member States regularly measure trends of populations of all listed species, to identify conservation priorities and to assess the efficiency of undertaken protection measures. Regular monitoring is also pivotal to identify threats. For instance, factors potentially driving population changes can be detected during monitoring programs, and this can allow to deliver effective and prompt conservation solutions [5, 6]. A continuous monitoring of conservation status and threats is particularly important for narrow endemic species that are more likely exposed to stochastic or directional threats than taxa with occurrences distributed over larger areas [7].

Monitoring programs are often limited by accessibility. Difficult access makes surveys more expensive and less feasible, thus the least accessible areas rarely are the focus of regular monitoring, and biodiversity surveys often remain concentrated in the most accessible areas [4, 8, 9]. Therefore, species living in inaccessible, remote areas often suffer insufficient monitoring, and declines or population changes might remain unnoticed. However, some of the areas with lowest accessibility also are very important for conservation. Roadless areas have a higher ecological value, given that some of them are among the most pristine ecosystems on Earth [10]. Furthermore, endemic species are particularly frequent in small islands and mountains [11–13], but these also are among the areas with lowest accessibility [8, 9, 14]. Improving biodiversity information in these remote areas is a conservation priority that is only seldom achieved.

**Fig 1:**
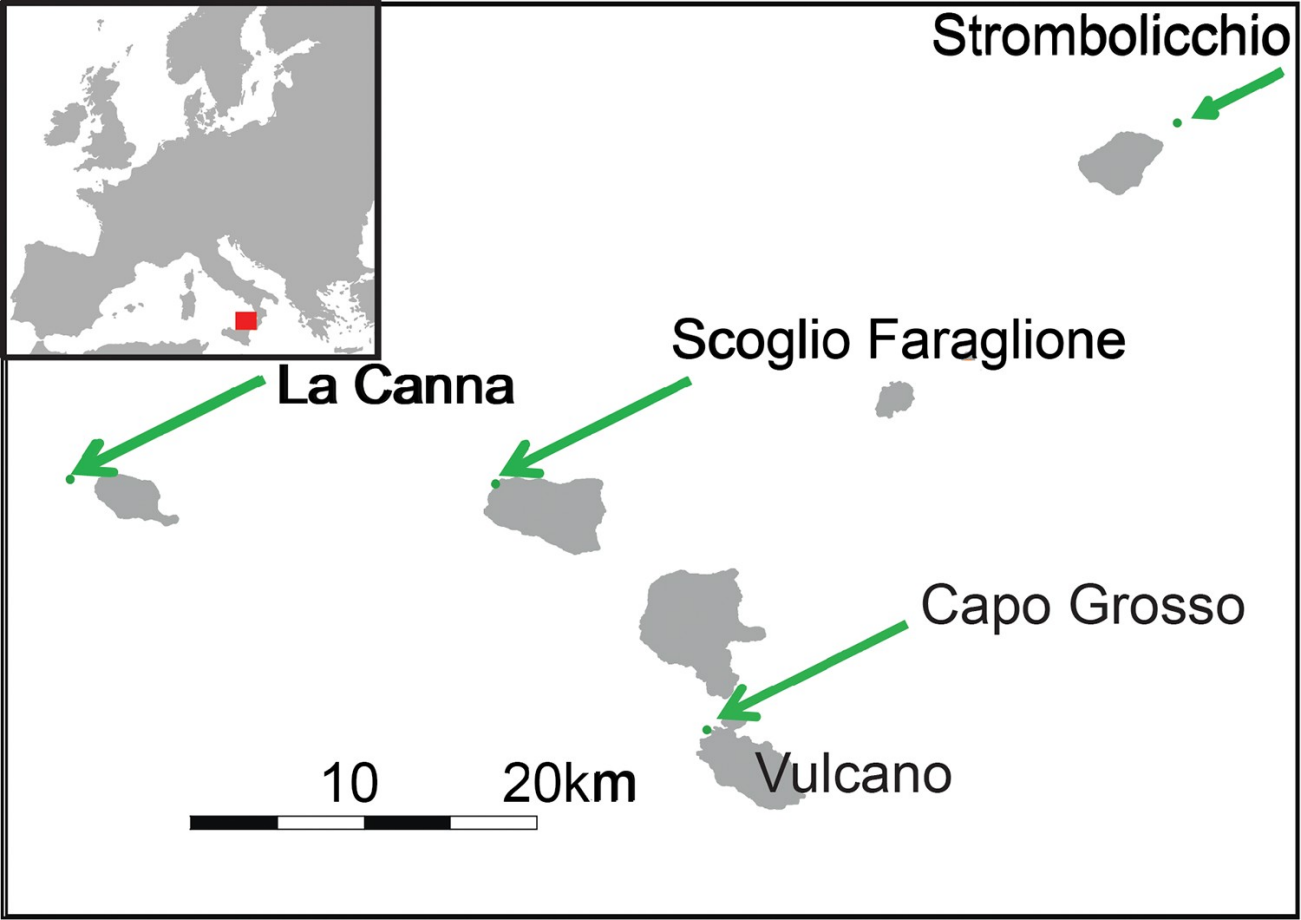
Distribution of the Aeolian wall lizard *Podarcis raffonei*. Distribution of the extant populations of *Podarcis raffonei*. The figure has been drawn by GFF on the basis of public domain shapefiles at http://www.naturalearthdata.com/.

Among the European vertebrates, the Aeolian wall lizard *Podarcis raffonei* (Mertens, 1952) is among the species with the smallest geographic distribution [15], and one of the most threatened by extinction [16]. This lizard is endemic of the Aeolian Archipelago in Southern Italy; currently, it is only reported from three tiny islets, and from a small peninsula of the Vulcano island [see methods, Fig 1 and Table 1; 16–18]. None of these areas is easily accessible. Despite some studies and reviews performed on this species [16, 18–23], a regular monitoring program is still lacking.

**Table 1:** Extent of occurrence and size of populations of *Podarcis raffonei*.

Site	Total area (m^2^)	Available area (m^2^)	Estimated population size	source
Punta Capo Grosso	6920	2990	975–1424	*N*-mixture models
			~1338 adults	removal sampling
Strombolicchio	3070	1600	500–700	Capula and Lo Cascio (25)
Scoglio Faraglione	4880	930	200–400	Lo Cascio (20)
La Canna	940	5*	83±53	Lo Cascio, Grita (21)

Total area and area available as habitat for lizards of the four remaining localities of *P*. *raffonei* and relative estimated population size (*: obtained from [21]).

Nevertheless, this lizard is considered to have suffered a dramatic decline during the last decades, and the IUCN Red List suggests a decreasing population trend [24]. Habitat loss, urbanization, wildfires, overgrazing, and inbreeding have all been proposed as potential driving factors [18, 19, 24–28]. It has been suggested that historical declines of *P*. *raffonei* might be related with the expansion of the Italian wall lizard *P*. *siculus* [18, 19], which has been introduced across the whole Mediterranean basin and adjacent areas, as well as in the UK and the US and Canada, by human activities [29, 30]. The competition with this introduced lizard is thus a possible driver of the decline of *P*. *raffonei* [28]. In fact, experimental evidence suggests that *P*. *siculus* is more aggressive, more exploratory, bolder, more neophilic and more efficient at finding and exploiting food resources than several native *Podarcis* species [31–33]. Furthermore, hybrids between *P*. *raffonei* and *P*. *siculus* have been observed in nature [34], suggesting that hybridization might be a further threat. Nevertheless, the causes of the decline of *P*. *raffonei* are not fully understood, because of the limited amount of data available for testing the proposed hypotheses. Finally, morphological identification of wall lizards is complicated by their high intraspecific diversity, both in shape and colouration, and by strong phenotypic plasticity, with some species showing significant changes of colour pattern across populations, ontogenically or throughout the year [35, 36].

Given the rapidity of the decline of *P*. *raffonei* in the Vulcano island, and the small size of extant populations, frequent monitoring is pivotal to assess the status of populations, to evaluate the driving factors and to identify prompt management strategies. The aim of this study was examining the fate of the Vulcano population, i.e. the population occupying the largest habitat patch where the Aeolian wall lizard still survives [28; see results]. We performed repeated surveys on this population, documenting shifts in the frequency of phenotypes, and providing the first insights on potential processes. Finally, through the analysis of individuals kept in captivity, we showed how individual phenotypes can significantly change through time, complicating assessments of temporal trends of the species.

## Methods

### Ethics statement

The study does not involve human or non-human primates. Lizards were manipulated according to international safety standard approved by the Italian Ministry for the Environment. Research activities were authorized by the Italian Ministry for the Environment (PNM 0004602, PNM 0008287, PNM 0008937).

### Study species

**Fig 2:**
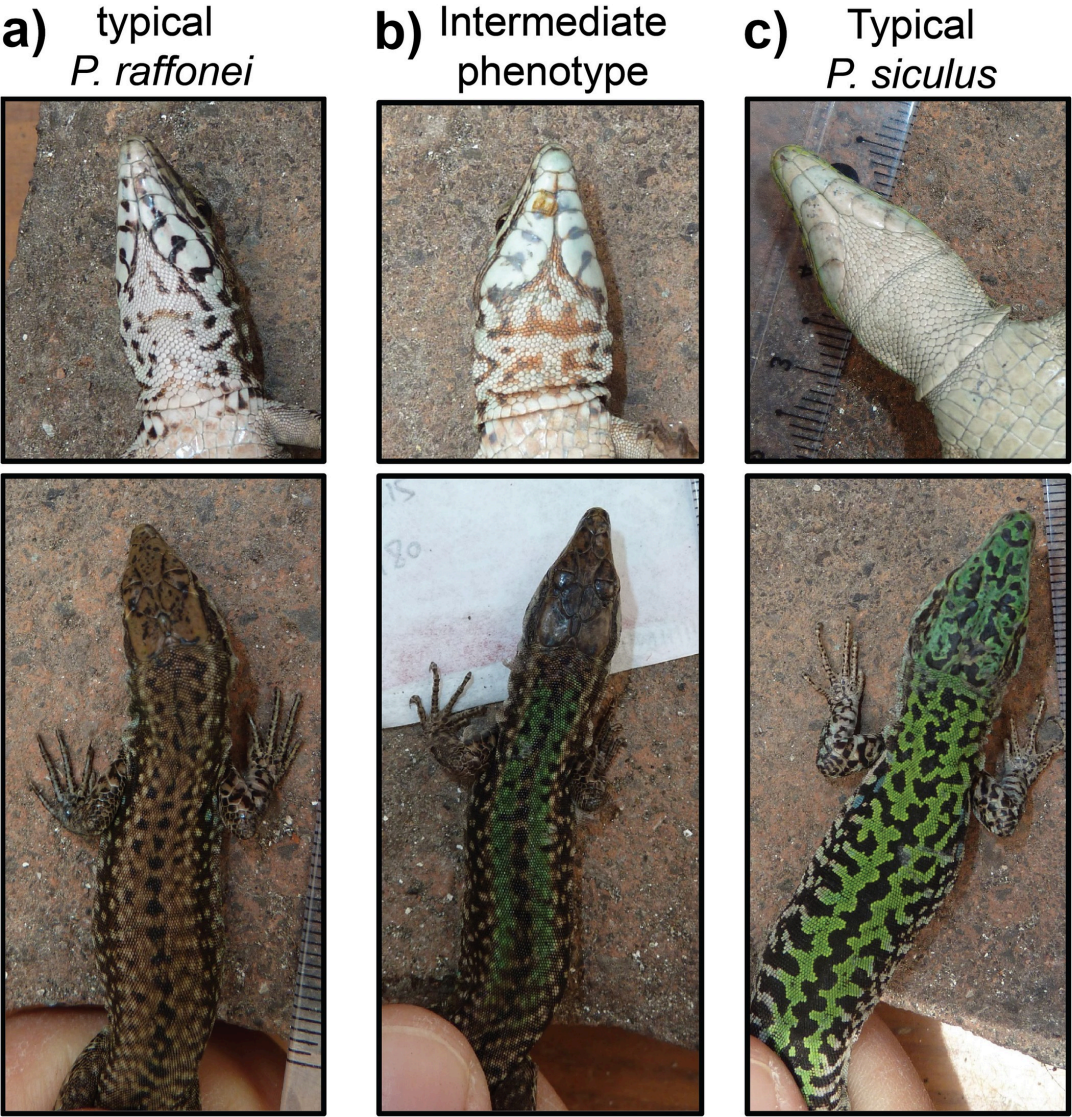
Typical colour patterns of *Podarcis raffonei* (a) and *P*. *siculus* (c), and patterns of individuals with the green phenotype, which is similar to the one of *P*. *siculus* (b).

The Aeolian wall lizard *Podarcis raffonei* is closely related to the Sicilian wall lizard *P*. *waglerianus* [37, 38] from which it has been separated on the basis of genetic divergence and separate ranges [39]. *Podarcis raffonei* is currently reported from only four localities within the Aeolian islands: three tiny islets (La Canna, Scoglio Faraglione and Strombolicchio), and the Capo Grosso peninsula of the Vulcano island [Fig 1, Table 1; 16–18, 28]. *Podarcis raffonei* is a medium-sized lizard, with a brown or dark-brown dorsal coloration. According to the species description in the "Fauna d’Italia", *P*. *raffonei* individuals can be identified by "the presence of evident dark markings on the throat (usually absent in *P*. *siculus*)"; colour of back generally brown with small dark dots and only occasionally olive hints, in males only [40] (Fig 2). The ventral coloration is white or red with dark gular spots [18, 25]. Individuals from the Vulcano island are described to have a snout-vent length (SVL) of adults of 55–68 mm [18, 25].

During the last decades, *Podarcis raffonei* has suffered a dramatic decline. In the 1950’s-1970’s, this lizard was abundant in several areas of the Vulcano island [18]. Capula and colleagues [19, 25] performed multiple surveys in these same areas during the period 1989–1999 and only found very few individuals of *P*. *raffonei* in two localities (0.5–1.6 individuals / ha), whereas the introduced Italian wall lizard, *P*. *siculus*, was abundant and widespread across the island [19, 25]. After 2000, almost no individuals of *P*. *raffonei* have been detected in Vulcano, with the exception of the Capo Grosso peninsula [18, 28] where the species was discovered in 1994 [18]. As a consequence, it has been suggested that populations outside the Capo Grosso peninsula might be "virtually extinct" [28]. Capo Grosso is a small (6,200 m^2^; roughly 100 x 50 m) peninsula, connected to the Vulcano island by a narrow isthmus with vertical cliffs, which makes the cape highly inaccessible and only weakly connected with the main island. Such isolation was assumed to hamper the colonization by *P*. *siculus*, so that until recent years *P*. *raffonei* was the only lacertid inhabiting the cape and its population was deemed to remain stable [18, 28].

### Species range, population size data, and housing

Information on the surface of the four localities with *P*. *raffonei* was calculated on the basis of the orthophotos of the Italian Geo-portal (http://www.pcn.minambiente.it/mattm/) (pictures taken on November 2015, resolution 0.5 m). The surface of the sites was used to calculate the extent of occurrence of the species (EOO, i.e. the surface of the species range). However, the lowest-altitude portions of all sites (< 10 m above sea level) are heavily washed by waves [20], and a relevant portion of them is occupied by bare rocks, particularly nearby sea shore, and lizards only rarely use it [20, 21]. We thus also calculated the surface where vegetation is present and / or the terrain is relatively flat, as these are the areas where lizards actually live [20, 21]. This was assumed to represent the surface of available habitat, and can be used to estimate the area of occupancy (AOO) of the species according to IUCN guidelines [7]. The surface of available habitat was calculated based on orthophotos and on polygons delimited by GPS points recorded in the field. For La Canna, we used the estimate of vegetated area by Lo Cascio, Grita (21).

During late summer of 2015 (end of August) and spring 2017 (end of April-beginning of May) we carried out field surveys in the Capo Grosso peninsula (38.4°N, 14.9°E) in order to estimate the size and the status of the last population of *P*. *raffonei* on Vulcano. In both occasions, adult lizards were captured by noosing, measured, sexed, and photographed. In 2015, only one capture session was performed (2 people, eight man-hours of capture), and individuals were immediately released. In 2017, five capture sessions were performed (2–6 people per session). Species identity of captured individuals was assessed on the basis of morphological features (Fig 2). *Podarcis raffonei* typically has a brown coloration and brown spots on the throat, while *P*. *siculus* has a uniform white throat and generally has extensive green in the back [40]. Previous studies using genetic protein markers suggest that hybrids between the two species have intermediate coloration (e.g., spotted throat but extensive green in the back) [34]. We also measured SVL of captured individuals and we used ANOVA to assess differences in SVL between sexes and phenotypes.

In 2017, we estimated the adult population size of the Capo Grosso population using two approaches: *N-*mixture models [41] and removal sampling. *N-*mixture models were performed on the basis of four linear transects, each repeated 2–3 times, where we performed visual encounter surveys and we counted the number of active adult lizards. Furthermore, all the captured males were temporally transported in terraria for behavioural and ecophysiological tests and for conservation purposes (unpublished data), thus it was possible using removal sampling [42] for an independent estimate of the total population size of adult males. Additional details on population size estimation are provided elsewhere [43]. Tail tips were collected and stored in pure ethanol for future genetic analyses.

A subset of individuals (N = 40, 20 males and 20 females) showing the typical brown coloration collected during the 2017 sessions was kept and maintained under captive-breeding conditions at the Fondazione Bioparco of Rome (former zoo) for conservation actions. Individuals were hosted in an outdoor facility designed and built especially for containing lizards, preventing their escape and the entry of possible predators or other lizard species form the outside. Spontaneous grasses were left free to grow inside the cages and were managed in order to maintain enough space for lizards to bask. The habitat inside the cages remained the same during captivity period. In winter, heating lamps were provided to simulate natural conditions (i.e., to prevent environmental temperature to go below 8°C as the minimum temperature recorded during winter in the native range of the species). Lizards were fed with crickets (*Acheta domesticus*) and water was supplied *ad libitum*. Pictures of the dorsal and ventral sides of the lizards were taken every 6–9 months to monitor possible colour changes due to seasonality, except during the 2020 lockdown related to the COVID-19 pandemics.

## Results

The Capo Grosso area represented 44% of the extent of occurrence of *P*. *raffonei*. In all the sites with *P*. *raffonei*, a large portion is occupied by bare rocks and / or are very close to the sea, particularly in the smallest islets. If only the core habitats were considered, the whole AOO of *P*. *raffonei* is reduced to 5,000 m^2^, with Capo Grosso representing 54% of it (Table 1).

### Phenotype and abundance of wild individuals

**Fig 3:**
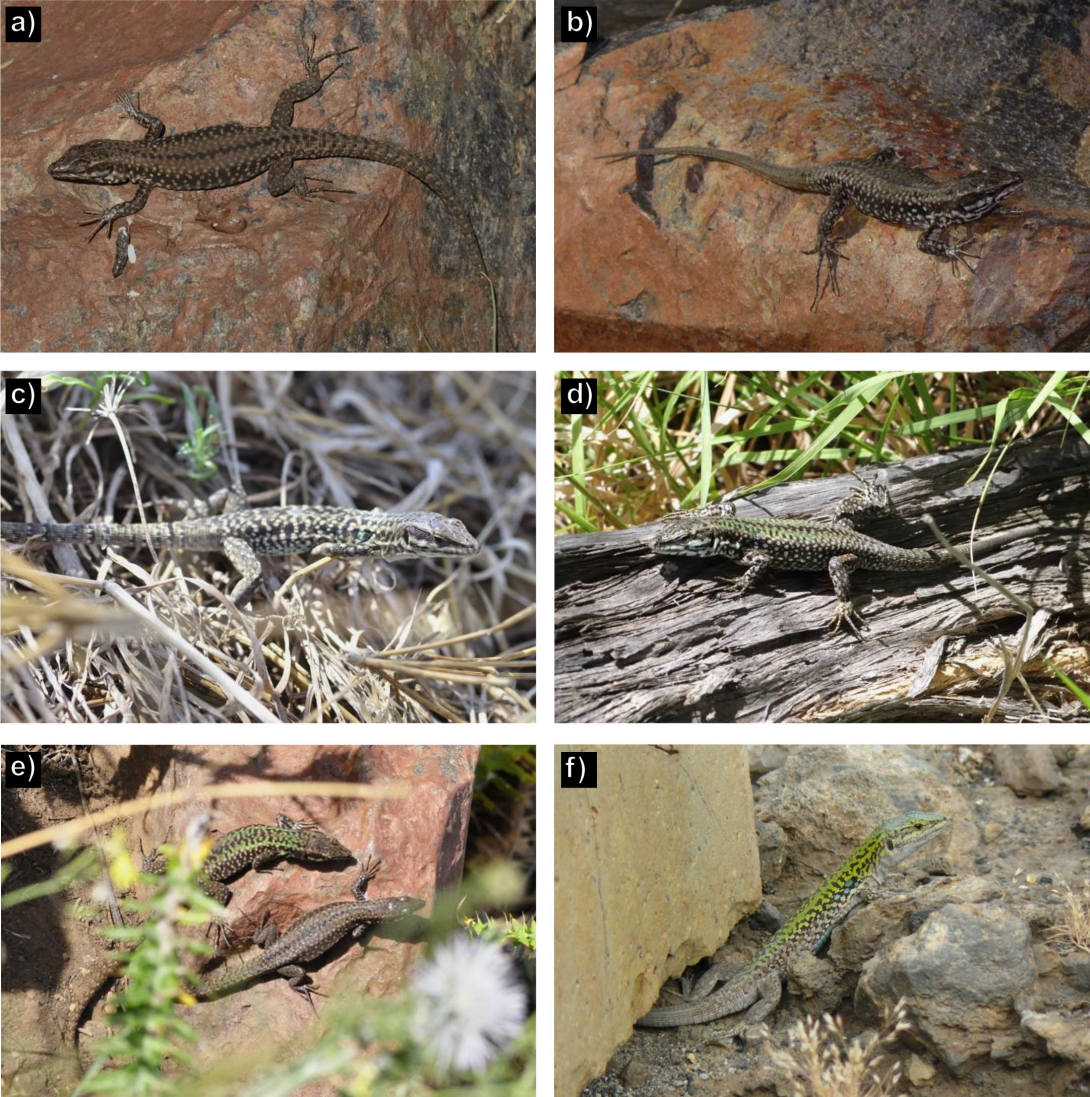
a, b): typical phenotype of *Podarcis raffonei* from Capo Grosso c, d): individuals with the green phenotype, showing the typical spotted throat but a green dorsal pattern similar to the phenotype of *P*. *siculus*. e): interactions between a male with a green phenotype and a female with typical *P*. *raffonei* phenotype; a few seconds later the female performed tongue flicking. f) typical phenotype of *P*. *siculus* on Vulcano (photos by G.F. Ficetola and D. Salvi).

In August 2015, thirty individuals were captured (17 males and 13 females) in approx. 8 man-hours of sampling (capture rate: 3.75 individuals / man-hour). All individuals showed the typical dorsal and ventral pattern of *P*. *raffonei* (Fig 2A) with the exception of one single male which showed a robust body and green hue on the dorsal pattern, while the ventral pattern with brown spots on the throat matched the typical features of *P*. *raffonei* (Fig 3C).

**Fig 4:**
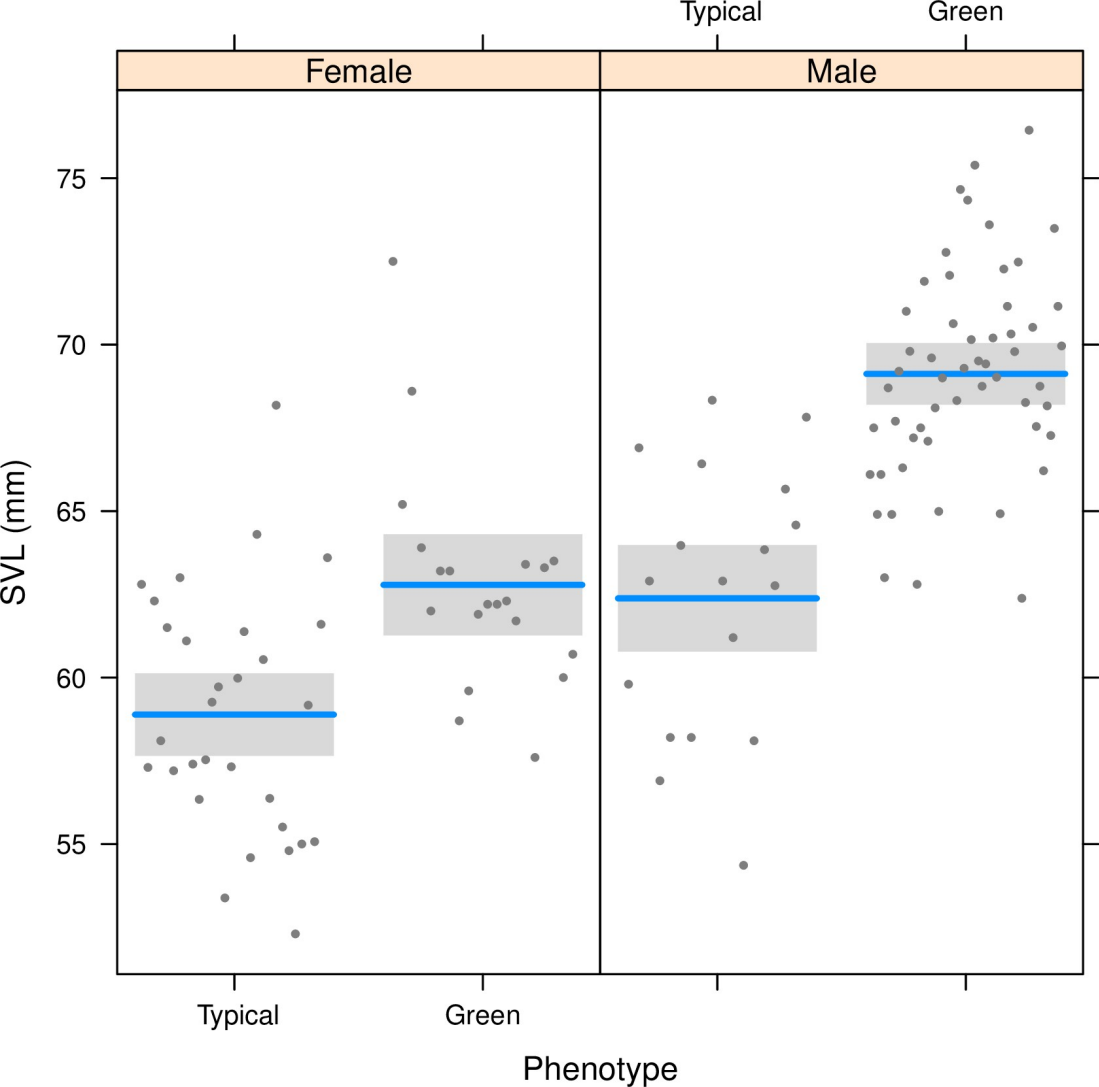
Differences in snout-vent length (SVL) between male and female lizards from Capo Grosso, and differences between individuals with the typical phenotype and individuals with the green phenotype. Shaded areas indicate 95% confidence intervals; dots represent the measured individuals.

In April-May 2017, we captured a total of 131 lizards in Capo Grosso. Average capture rate was 3.3 individuals / man-hour. Out of the 131 individuals, 63 were males and 68 females. Within males, 24% showed the typical dorsal and ventral pattern of *P*. *raffonei*, whereas 76% of individuals showed some features that do not match the typically described phenotype of *P*. *raffonei*. These individuals showed a robust body, green dorsal pattern, white or pale orange ventral pattern with dark spots on the throat (e.g., Fig 2B; hereafter: green phenotype; Fig 3). Within females, 69% showed the typical brown dorsal pattern, while in 31% of individuals showed the green phenotype. Individuals with the green phenotype were found across the whole peninsula; no individual showed the phenotype of pure *P*. *siculus* individuals. Males showed significantly larger SVL than females (ANOVA: *F*_1,118_ = 57.2, *P* < 0.001). Individuals with the green phenotype were larger than the brown ones (*F*_1,118_ = 63.3, *P* < 0.001); furthermore, a significant interaction between sex and phenotype suggested that the size difference between individuals with the typical brown phenotype and the green phenotype are particularly strong for males (*F*_1,118_ = 4.4, *P* = 0.039; Fig 4).

During visual encounter surveys performed in replicated transects in 2017, we detected 85 lizards. The total population size estimated by *N*-mixture models was 1050 individuals (95% CI: 847–1280). The removal method estimated a total population size of 538 males. The number of captured males (63) was small compared to the total population size estimated by the two models, thus confidence intervals for this estimate are not available. Assuming a balanced sex ratio, as generally observed for *P*. *raffonei* [25], this would result in a total abundance of adult of approx. 1000 / 1100 individuals. Complete details on the estimated population size, and discussion of their limitations, are provided elsewhere [43].

### Phenotype of housed individuals

**Fig 5:**
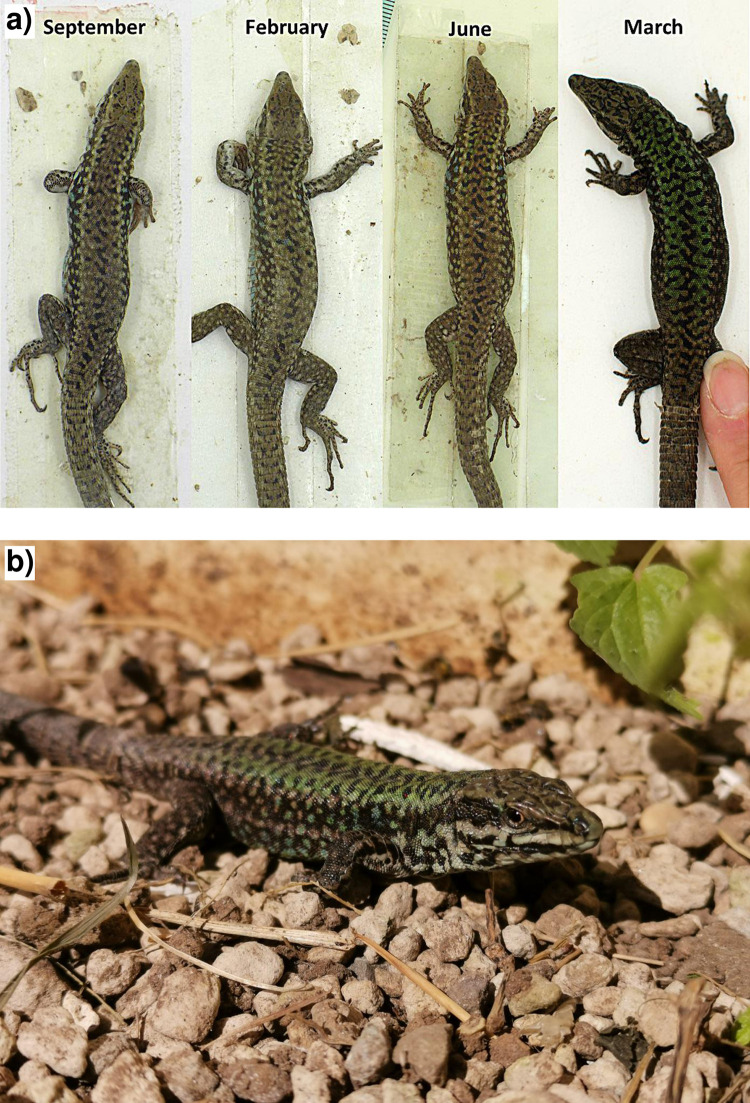
Phenotypes of one captive male *Podarcis raffonei* in different months (photos by L. Vignoli).

Forty individuals with the typical brown coloration were captured and maintained under captive conditions for conservation purposes. All the 40 lizards were brown in September 2017 at the first assessment after the capture, and were brown or greyish in February 2018, June 2018, and December 2019. In March 2021, all the 13 surviving individuals (two males and 11 females) showed the green phenotype (Fig 5).

## Discussion

The Aeolian wall lizard, *Podarcis raffonei* is probably the European reptile with the smallest range (<2 ha). Within such a small range, the Capo Grosso peninsula has a key conservation importance for this species. First, Capo Grosso is the largest site of known occurrence of *P*. *raffonei* (Table 1) and represents more than 56% of the whole habitat available for the species. So, at least until 2015, Capo Grosso presumably hosted most of the extant individuals of *P*. *raffonei*. Furthermore, this was the only population not limited to a tiny, isolated islet, therefore it might have suffered less genetic drift and / or inbreeding, compared to the other three (microinsular) populations. As such, the population in Capo Grosso is expected to retain a significant portion of the evolutionary potential of the species [44]. Our data suggest that in 2017 the Capo Grosso population hosted approx. 800–1300 adult lizards [43]. The capture rate during 2015 (3.75 individuals / man-hour) was similar or slightly higher than the capture rate during 2017. Therefore, a population size above 1,000 individuals was likely also present in 2015. The total size of the other three populations, summing the values of the three localities, has been estimated to be 780–1200 individuals (Table 1). Surveys performed in 1989–1999 detected *P*. *raffonei* also in two other areas of the Vulcano island (Capula, 1993, 1994; Capula et al., 2002), i.e. Vulcanello and Gran Cratere (in the northern and central part of Vulcano, respectively), but more recent surveys based on morphological identification did not confirm the occurrence of *P*. *raffonei* in these areas, and only detected the invasive *P*. *siculus* [28]. Thus overall, data on habitat surface and population estimates supported the idea that, until 2015, Capo Grosso was by far the largest *P*. *raffonei* population, accounting for approx. 50% of all the individuals of this species.

While the total number of lizards estimated to occur on Capo Grosso has roughly remained stable from 2015 to 2017, the frequency of individuals with the typical *P*. *raffonei* phenotype has dramatically changed in this two-year period. In 2015 we only detected one single male with the green dorsal pattern. Similarly, D’Amico, Bastianelli (28) surveyed the Capo Grosso population in March 2016, and only reported the occurrence of *P*. *raffonei*. Conversely, in 2017 approx. 50% of individuals did not show the typical *P*. *raffonei* features. The morphological features of these individuals match the description of hybrids between these two species [25, 34]. Some intermediate phenotypes between *P*. *raffonei* and *P*. *siculus* observed by Capula in Vulcano were confirmed to be hybrids based on allozyme analyses [34]. However, introgression patterns of *Podarcis* lizards can be extremely complex [45], and the identification of these lizards is complicated by strong phenotypic plasticity [35], therefore genetic data should be combined with phenotype data to confirm the hypothesis of hybridization and to characterize introgression patterns.

If the phenotype alone is used to estimate the actual frequency of *P*. *raffonei*, it seems that the recent arrival of *P*. *siculus*-like individuals in Capo Grosso has placed at risk the population of *P*. *raffonei* from this site, with a 50% decline of individuals with the typical phenotype in less than two years. However, the interpretation of these data is complicated by the strong phenotypic plasticity observed in some of the captured individuals, which showed the typical brown phenotype in August 2017 and a green phenotype in March 2021. Several non-exclusive hypotheses can be formulated on the processes driving the changes in the in the frequency of *P*. *raffonei*-like phenotypes on Capo Grosso. These include decline of *P*. *raffonei* due to habitat change and competitive exclusion by *P*. *siculus*, or, instead, plasticity in colour pattern of *P*. *raffonei*. Habitat remained similar between 2015 and 2017, therefore, it is unlikely that these changes are determined by habitat modifications as suggested for the observed decline of the species in the main island of Vulcano [34]. Competition with the invasive *P*. *siculus* possibly also involving hybridization, and phenotypic plasticity of *P*. *raffonei* are the two most likely explanations of the observed phenotype variation [46, 47]. The hypothesis of competitive exclusion of the native populations of *P*. *raffonei* by *P*. *siculus* was formulated by Capula [34, 39] to explain the widespread occurrence of *P*. *siculus* in the Aeolian Archipelago, following its introduction, and the relictual distribution of *P*. *raffonei* in three tiny islets and a small portion of the Vulcano island. In the same studies, introgressed allele of *P*. *raffonei* were detected in 25% of the analyzed individuals of *P*. *siculus* on Lipari and a high percentage (~15%) of hybrids between the two species was found on Vulcano, suggesting that also hybridization with *P*. *siculus* might have played a role in the extinction of *P*. *raffonei* from Lipari and in the observed decline on Vulcano [34, 39]. Hybridization with alien species is a serious threat for endemic species in small islands, since it can cause characters to be unequally transferred from the alien to the endemic species.

Phenotypic plasticity of coloration in lacertid lizards is an understudied topic [35, 48]. While seasonal variation has been reported for *P*. *bocagei*, *P*. *siculus* and *P*. *waglerianus* [35, 49, 50], it has never been reported for *P*. *raffonei*. Individuals of *P*. *raffonei* from Vulcano island have always been described with brown coloration [25]. However, by maintaining individuals in captive breeding conditions for four years, we discovered that *P*. *raffonei* can show coloration shifts through time, and adults could have a green pattern during spring that make them extremely similar to previously described hybrids between *P*. *raffonei* and *P*. *siculus* [25, 34]. Therefore, phenotypic plasticity of resident *P*. *raffonei* can also explain the significant change in the relative frequency of brown (*P*. *raffonei*-like) and green (*P*. *siculus*-like) phenotypes observed at Capo Grosso during the study period. We highlight that the morphological identification of these lizards has always been extremely challenging. In fact, populations currently belonging to *P*. *raffonei* were initially described as subspecies of *P*. *siculus* (populations from La Canna and Scoglio Faraglione) or *P*. *waglerianus* (populations from Strombolicchio and Vulcano), and only subsequent genetic analyses clarified that these populations did not belong to either of the two species, and instead they represent a distinct species [25, 34, 37, 39]. This is actually not unusual in wall lizards, as in the last decade the implementation of genetic analyses is showing the existence of a very large number of cryptic species for which no diagnostic morphological characters are available, and for which the identification in the field is challenging due to wide overlap of intra- and interspecific phenotypic variation [51–53].

The hypotheses of competition/hybridization with invasive lizards and phenotypic plasticity are not mutually exclusive. It is thus possible that both plasticity in colour pattern and hybridization with *P*. *siculus* jointly played in determining the phenotypic changes observed in the population of *P*. *raffonei* of Capo Grosso. In order to disentangle between these processes and to evaluate their relative contribution, a comprehensive genomic assessment of these individuals is currently in progress, with the aim of providing an accurate assessment on the occurrence of hybridization and understanding the evolutionary processes underlying the occurrence of individuals with ‘green’ phenotypes in Capo Grosso. Such a genetic assessment requires the analysis of a very large number of loci sampled across the whole genome, given that a limited number of markers does not allow to correctly quantify introgression in recently admixed populations [54], and even small portions of the genome can be responsible of strong variation at both morphological, ecophysiological and reproductive traits [45, 55, 56].

In conclusion, this study documents the complexity of assessing long-term trends in microendemic species living in poorly accessible areas, particularly when they show complex patterns of intraspecific variation and plasticity. While robust genomic evidence takes time to be, a simple principle of caution recommends immediate management actions and of standardized monitoring protocols. Accurate genomic screening of populations to identify areas where *P*. *raffonei* individuals are actually present is urgently needed. Furthermore, the removal of *P*. *siculus* is potentially a useful strategy, but is complicated by the challenges of species identification in the field, and by the difficulty of capturing lizards with approaches that do not harm the native fauna in a very complex landscape where lizards can easily escape. Without a clear conservation strategy, this population may become extinct in a few generations and, given that *P*. *siculus* has already been introduced into multiple islets [57], the same fate might also occur to the small populations living in more isolated sites. The scientific community and the biodiversity managers should thus implement immediate measures to avoid the extinction of the last survivors of the Vulcanian lineage of *P*. *raffonei*, but also identify long-term strategies to allow the persistence of this endemic species in the Aeolian Archipelago.

## References

[1] Yoccoz NG, Nichols JD, Boulinier T. Monitoring of biological diversity in space and time. Trends Ecol Evol. 2001;16(8):446–53. doi: 10.1016/s0169-5347(01)02205-4 .

[2] Reynolds JH, Thompson WL, Russell B. Planning for success: Identifying effective and efficient survey designs for monitoring. Biol Conserv. 2011;144:1278–84. doi: 10.1016/j.biocon.2010.12.002 .

[3] Ficetola GF, Romano A, Salvidio S, Sindaco R. Optimizing monitoring schemes to detect trends in abundance over broad scales. Anim Conserv. 2018;21:221–31. doi: 10.1111/acv.12356

[4] Marta S, Lacasella F, Romano A, Ficetola GF. Cost-effective spatial sampling designs for field surveys of species distribution. Biodivers Conserv. 2019;28:2891–908 10.1007/s10531-019-01803-x.

[5] Nichols JD, Williams BK. Monitoring for conservation. Trends Ecol Evol. 2006;21(12):668–73. doi: 10.1016/j.tree.2006.08.007 . 16919361

[6] Purse BV, Golding N. Tracking the distribution and impacts of diseases with biological records and distribution modelling. Biol J Linn Soc. 2015;115:664–77. doi: 10.1111/bij.12567

[7] IUCN Standards and Petitions Subcommittee. Guidelines for Using the IUCN Red List Categories and Criteria. Version 12. http://www.iucnredlist.org/documents/RedListGuidelines.pdf: IUCN; 2016.

[8] Ficetola GF, Bonardi A, Sindaco R, Padoa-Schioppa E. Estimating patterns of reptile biodiversity in remote regions. J Biogeogr. 2013;40:1202–11.

[9] Yang W, Ma K, Kreft H. Environmental and socio-economic factors shaping the geography of floristic collections in China. Global Ecol Biogeogr. 2014;23:1284–92. doi: 10.1111/geb.12225

[10] Ibisch PL, Hoffmann MT, Kreft S, Pe’er G, Kati V, Biber-Freudenberger L, et al. A global map of roadless areas and their conservation status. Science. 2016;354(6318):1423. doi: 10.1126/science.aaf7166 27980208

[11] Rosauer DF, Jetz W. Phylogenetic endemism in terrestrial mammals. Global Ecol Biogeogr. 2015;24:168–79. doi: 10.1111/geb.12237

[12] Lamoreux JF, Morrison JC, Ricketts TH, Olson DM, Dinerstein E, McKnight MW, et al. Global tests of biodiversity concordance and the importance of endemism. Nature. 2006;440:212–4. doi: 10.1038/nature04291 16382239

[13] Mittermeier RA, Gil PR, Hoffman M, Pilgrim J, Brooks T, Mittermeier CG, et al. Hotspots revisited. Mexico City: CEMEX; 2004.

[14] Ficetola GF, Cagnetta M, Padoa-Schioppa E, Quas A, Razzetti E, Sindaco R, et al. Sampling bias inverts ecogeographical relationships in island reptiles. Global Ecol Biogeogr. 2014;23:1303–13.

[15] Sillero N, Campos J, Bonardi A, Corti C, Creemers R, Crochet PA, et al. Updated distribution and biogeography of amphibians and reptiles of Europe based on a compilation of countrywide mapping studies. Amphibia-Reptilia. 2014;35:1–31. doi: 10.1163/15685381-00002935

[16] Gippoliti S, Capula M, Ficetola GF, Salvi D, Andreone F. Threatened by Legislative Conservationism? The Case of the Critically Endangered Aeolian Lizard. Frontiers in Ecology and Evolution. 2017;5:130. doi: 10.3389/fevo.2017.00130

[17] Capula M, Lo Cascio P. *Podarcis raffonei* (Mertens, 1953). In: Sindaco R, Doria G, Razzetti E, Bernini F, editors. Atlas of Italian Amphibians and Reptiles. Firenze: Polistampa; 2006. p. 480–5.

[18] Lo Cascio P. Attuali conoscenze e misure di conservazione per le popolazioni relitte dell’endemica lucertola delle Eolie, *Podarcis raffonei* (Squamata Sauria). Naturalista siciliano, ser 4. 2010;34:295–317.

[19] Capula M, Luiselli L, Bologna MA, Ceccarelli A. The decline of the Aeolian wall lizard, *Podarcis raffonei*: causes and conservation proposals. Oryx. 2002;36:66–72. .

[20] Lo Cascio P. Aspetti ecologici e problemi di conservazione di una popolazione di Podarcis raffonei (Mertens, 1952) (Reptilia: Lacertidae). Naturalista siciliano, ser 4. 2006;30:495–521.

[21] Lo Cascio P, Grita F, Guarino L, Speciale C. A little is better than none: new insights into the natural history of the Aeolian wall lizard Podarcis raffonei from La Canna stack (Squamata Sauria). Naturalista siciliano, ser 4. 2014;38:355–66.

[22] Capula M, Corti C, Lo Cascio P, Luiselli L. Termal ecology of the Aeolian wall lizard, *Podarcis raffonei*. What about body temperatures in microinsular lizards? In: Capula M, Corti C, editors. Scripta Herpetologica Studies on Amphibians and Reptiles in honour of Benedetto Lanza. Latina, Italy: Edizioni Delverde; 2014. p. 39–47.

[23] Caruso Y, Macale D, Luiselli L, Vignoli L. Thermoregulation comparisons between a threatened native and an invasive lizard species. Herpetol J. 2021;31:70–6.

[24] Corti C, Pérez-Mellado V, Sindaco R, Romano A. Podarcis raffonei. IUCN Red List of Threatened Species: IUCN. Available at www.iucnredlist.org; 2009. p. 10.2305/IUCN.UK.009.RLTS.T61552A12514822.en.

[25] Capula M, Lo Cascio P. *Podarcis raffonei* (Mertens, 1952). In: Corti C, Capula M, Luiselli L, Razzetti E, Sindaco R, editors. Fauna d’Italia, Reptilia. Bologna: Edizioni Calderini de Il Sole 24 ORE; 2011. p. 401–7. doi: 10.1001/jama.2011.207

[26] Lo Cascio P, Ficetola GF. *Podarcis raffoneae* (Mertens, 1952) (Lucertola delle Eolie). In: Stoch F, Genovesi P, editors. Manuali per il monitoraggio di specie e habitat di interesse comunitario (Direttiva 92/43/CEE) in Italia: specie animali. Roma: ISPRA; 2016. p. 280–1.

[27] Capula M. Low genetic variation in a critically endangered mediterranean lizard: conservation concerns for *Podarcis raffonei* (Reptilia, Lacertidae). Ital J Zool. 2004;71, Suppl. 1:161–6.

[28] D’Amico M, Bastianelli G, Faraone FP, Lo Valvo M. The Spreading of the Invasive Italian Wall Lizard on Vulcano, the Last Island Inhabited by the Critically Endangered Aeolian Wall Lizard. Herpetol Conserv Biol. 2018;13:146–57.

[29] Silva-Rocha I, Salvi D, Harris DJ, Freitas S, Davis C, Foster J, et al. Molecular assessment of *Podarcis sicula* populations in Britain, Greece and Turkey reinforces a multiple-origin invasion pattern in this species. Acta Herpetol. 2014;9(2):253–8. .

[30] Silva-Rocha I, Salvi D, Carretero MA, Ficetola GF. Alien reptiles on Mediterranean islands: a model for invasion biogeography. Divers Distrib. 2019;22:995–1005.

[31] Damas-Moreira I, Riley JL, Harris DJ, Whiting MJ. Can behaviour explain invasion success? A comparison between sympatric invasive and native lizards. Anim Behav. 2019;151:195–202. doi: 10.1016/j.anbehav.2019.03.008

[32] Damas-Moreira I, Oliveira D, Santos JL, Riley JL, Harris DJ, Whiting MJ. Learning from others: an invasive lizard uses social information from both conspecifics and heterospecifics. Biol Lett. 2018;14(10):20180532. doi: 10.1098/rsbl.2018.0532 30333265 PMC6227858

[33] Downes S, Bauwens D. An experimental demonstration of direct behavioural interference in two Mediterranean lacertid lizard species. Anim Behav. 2002;63(6):1037–46. doi: 10.1006/anbe.2002.3022

[34] Capula M. Natural hybridization in *Podarcis sicula and P*. *wagleriana* (Reptilia: Lacertidae). Biochem Syst Ecol. 1993;21(3):373–80. doi: 10.1016/0305-1978(93)90028-P

[35] Pellitteri-Rosa D, Gazzola A, Todisco S, Mastropasqua F, Liuzzi C. Lizard colour plasticity tracks background seasonal changes. Biol Open. 2020;9(6):bio052415. doi: 10.1242/bio.052415 . 32414767 PMC7286296

[36] Piras P, Salvi D, Ferrara G, Maiorino L, Delfino M, Pedde L, et al. The role of post-natal ontogeny in the evolution of phenotypic diversity in Podarcis lizards. J Evol Biol. 2011;24(12):2705–20. doi: 10.1111/j.1420-9101.2011.02396.x 21954968

[37] Salvi D, Pinho C, Mendes J, Harris DJ. Fossil-calibrated time tree of *Podarcis* wall lizards provides limited support for biogeographic calibration models. Mol Phylogenet Evol. 2021;161:107169. doi: 10.1016/j.ympev.2021.107169 33798673

[38] Yang W, Feiner N, Pinho C, Kaliontzopoulou A, While GM, Harris DJ, et al. Extensive introgression and mosaic genomes of Mediterranean endemic lizards. Nat Commun. 2021;in press. doi: 10.1038/s41467-021-22949-9 33980851 PMC8114931

[39] Capula M. Genetic variation and differentiation in the lizard, *Podarcis wagleriana* (Reptilia: Lacertidae). Biol J Linn Soc. 1994;52:177–96.

[40] Corti C, Capula M, Luiselli L, Razzetti E, Sindaco R. Fauna d’Italia, vol. XLV, Reptilia. Bologna: Calderini; 2011.

[41] Royle JA. *N*-mixture models for estimating population size from spatially replicated counts. Biometrics. 2004;60:108–15. doi: 10.1111/j.0006-341X.2004.00142.x 15032780

[42] Chao A, Chang SS. An estimating function approach to the inference of catch-effort models. Environmental and Ecological Statistics. 1999;6:313–34.

[43] Ficetola GF, Barzaghi B, Melotto A, Muraro M, Lunghi E, Canedoli C, et al. N-mixture models reliably estimate the abundance of small vertebrates. Sci Rep. 2018;8:10357. doi: 10.1038/s41598-018-28432-8 29985399 PMC6037707

[44] Frankham R, Ballou JD, Briscoe DA. Introduction to conservation genetics. Cambridge, UK: Cambridge University Press; 2002. doi: 10.1016/s0031-9384(02)00783-7

[45] Yang W, While GM, Laakkonen H, Sacchi R, Zuffi MAL, Scali S, et al. Genomic evidence for asymmetric introgression by sexual selection in the common wall lizard. Mol Ecol. 2018;27(21):4213–24. doi: 10.1111/mec.14861 30192998

[46] Rhymer JM, Simberloff D. Extinction by hybridization and introgression. Annu Rev Ecol Syst. 1996;27:83–109. doi: 10.1146/annurev.ecolsys.27.1.83 .

[47] Todesco M, Pascual MA, Owens GL, Ostevik KL, Moyers BT, Hubner S, et al. Hybridization and extinction. Evol Appl. 2016;9(7):892–908. doi: 10.1111/eva.12367 . 27468307 PMC4947151

[48] Cuervo JJ, Belliure J. Exploring the function of red colouration in female spiny-footed lizards (Acanthodactylus erythrurus): patterns of seasonal colour change. Amphibia-Reptilia. 2013;34(4):525–38. doi: 10.1163/15685381-00002912

[49] Faraone FP, Lo Valvo M. Seasonal variation in colour of the Sicilian wall lizard *Podarcis wagleriana*. 6° Congresso Nazionale della Societas Herpetologica Italica, Abstract Book. Roma: Societas Herpetologica Italica; 2006.

[50] Galan P. Cambios estacionales de coloración y comportamiento agonístico, de cortejo y de apareamiento en el lacértido *Podarcis bocagei*. Revista Española de Herpetología. 1995;9:57–75.

[51] Psonis N, Antoniou A, Kukushkin O, Jablonski D, Petrov B, Crnobrnja-Isailović J, et al. Hidden diversity in the Podarcis tauricus (Sauria, Lacertidae) species subgroup in the light of multilocus phylogeny and species delimitation. Mol Phylogenet Evol. 2017;106:6–17. doi: 10.1016/j.ympev.2016.09.007 27640951

[52] Kaliontzopoulou A, Carretero MA, Llorente GA. Morphology of the *Podarcis* wall lizards (Squamata: Lacertidae) from the Iberian Peninsula and North Africa: patterns of variation in a putative cryptic species complex. Zoological Journal of the Linnean Society. 2012;164(1):173–93. doi: 10.1111/j.1096-3642.2011.00760.x

[53] Kaliontzopoulou A, Pinho C, Harris DJ, Carretero MA. When cryptic diversity blurs the picture: a cautionary tale from Iberian and North African Podarcis wall lizards. Biol J Linn Soc. 2011;103(4):779–800. doi: 10.1111/j.1095-8312.2011.01703.x.

[54] Della Croce P, Poole GC, Luikart G. Detecting and quantifying introgression in hybridized populations: simplifying assumptions yield overconfidence and uncertainty. Mol Ecol Resour. 2016;16(6):1287–302. doi: 10.1111/1755-0998.12520 26946238

[55] Yang W, Feiner N, Laakkonen H, Sacchi R, Zuffi MAL, Scali S, et al. Spatial variation in gene flow across a hybrid zone reveals causes of reproductive isolation and asymmetric introgression in wall lizards. Evolution. 2020;74(7):1289–300. doi: 10.1111/evo.14001 32396671

[56] Andrade P, Pinho C, Pérez i de Lanuza G, Afonso S, Brejcha J, Rubin C-J, et al. Regulatory changes in pterin and carotenoid genes underlie balanced color polymorphisms in the wall lizard. Proceedings of the National Academy of Sciences. 2019:201820320. doi: 10.1073/pnas.1820320116 30819892 PMC6431182

[57] Lo Cascio P, Corti C. The micro-insular distribution of the genus *Podarcis* within the Aeolian Archipelago: historical vs. palaeogeographical interpretation. In: Corti C, Lo Cascio P, Biaggini M, editors. Mainland and insular lacertid lizards: a mediterranean perspective. Firenze: Firenze University Press; 2006. p. 91–102.

